# Supplementary material for: Splice-Junction-Based Mapping of Alternative Isoforms in the Human Proteome
Source: Cell Rep. Author manuscript; Available in PMC 2020 Jan 15. (PMC6961840; doi:10.1016/j.celrep.2019.11.026)
Supplement: 2 [file NIHMS1546469-supplement-2.zip › DF1/PXD009737/TestisLysC/TestisLysC_34_SEC24C_RIDPDAIPSPIQVIEDDRNNRGTEPFVTGVRGQVPPLVTTNFLVK.pdf]

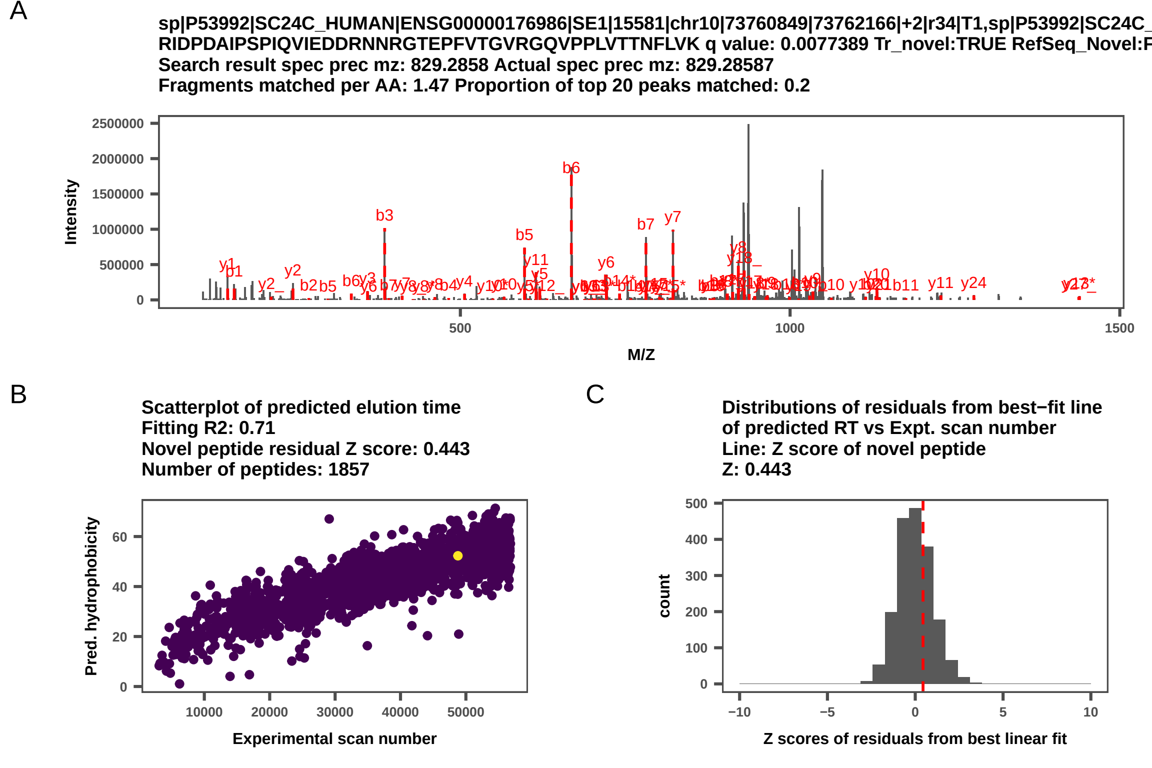
A
sp|P53992|SC24C_HUMAN|ENSG00000176986|SE1|15581|chr10|73760849|73762166|+2|r34|T1,sp|P53992|SC24C_
RIDPDAIPSPIQVIEDDRNNRGTEPFVTGVRGQVPPLVTTNFLVK q value: 0.0077389 Tr_novel:TRUE RefSeq_Novel:F
Search result spec prec mz: 829.2858 Actual spec prec mz: 829.28587
Fragments matched per AA: 1.47 Proportion of top 20 peaks matched: 0.2
Intensity
2500000
2000000
1500000
1000000
500000
0
b6
b3
b7
y7
b5
y8
y18_
y11
y6
y5
y1
b1
y2
y10
y11
y24
b11
M/Z
500
1000
1500
B
Scatterplot of predicted elution time
Fitting R2: 0.71
Novel peptide residual Z score: 0.443
Number of peptides: 1857
Pred. hydrophobicity
60
40
20
0
10000
20000
30000
40000
50000
Experimental scan number
C
Distributions of residuals from best−fit line
of predicted RT vs Expt. scan number
Line: Z score of novel peptide
Z: 0.443
count
500
400
300
200
100
0
−10
−5
0
5
10
Z scores of residuals from best linear fit
